# Morbid Growths

**Published:** 1895-12-21

**Authors:** 


### PROGRESS IN SURGERY.

### MORBID GROWTHS.

**Tumours of the Breast.**—Mr. Mitchell Banks[1] discusses the value of certain signs and symptoms in cancer of the breast. He thinks that usually a period of one to two years elapses between the commencement of the disease and the onset of any pain in the part; and that an early occurrence of pain would militate against the idea of the disease being cancerous. Retraction of the nipple, he points out, must not be expected when the cancer is peripheral, but will certainly be present if the growth is situated in the centre of the breast. Mr. Banks has always been a strong advocate for thorough clearance of the axilla, whether enlarged glands can be felt or not, and he states that when a cancer of the breast has existed for twelve or eighteen months it may be taken as a certainty that the glands are affected. He records two cases to show after what a lengthy period the disease may recur in the axillary glands. In one the breast was removed in America ten years before she came under his care. There was no trace of recurrence in the region of the scar, but the disease began to recur in the axillary glands. In the other the disease recurred in the glands of the axilla nine years after removal of the breast and most of the axillary glands.

Mr. Arbuthnot Lane[2] describes a new method of treating very extensive malignant disease of the breast. In cases where the skin is extensively involved and a large area has to be removed, he thinks there is a risk of septic infection, and that even if the surgeon succeeds in grafting such a large area the resulting scar will be a most unsatisfactory one. He therefore proposes to amputate the upper limb at the shoulder joint (as it will become useless from interference with its veins if the disease is not removed) after making from it a large flap, which is applied to cover the area from which the infected skin and breast have been removed. The flap would consist of the skin and subcutaneous tissues, with a part of the deltoid muscle at its base. The middle of the clavicle is first removed, and the subclavian artery tied. Then the glands in the subclavian triangle are removed, together with the axillary glands, and if necessary the pectoral muscles also. Mr. Lane has successfully performed the operation in one case.

**Erysipelas Toxins and Serum in Malignant Growths.**—Kopfstein[3] inoculated sheep with erysipelas streptococci and injected the serum from the animals in thirteen cases of carcinoma, one of sarcoma, and one of malignant lymphoma. The injections were followed by severe headache and pain in the back, with rigors and cyanosis, and the temperature rose several degrees. Violent burning pain in the tumour followed, and there was prolonged swelling and tenderness after the injection, and an abscess formed in one case. In another the tumour broke down and discharged carcinomatous masses without pus. In ulcerated growths the base took on a healthy action. Kopfstein thinks the action purely local, the only change taking place at the spot where the injection was made. He does not consider the method of any value.

Salvati and Gaetano[4] inoculated (or tried to inoculate) a horse with an infusion of triturated sarcoma by injecting it into the trachea; and then injected serum from the animal into persons suffering from cancer, but without benefit.

Czerny[5] has been trying Coley's method of injecting into the malignant growths the mixed toxines of erysipelas streptococci and bacillus prodigiosus. In one case—a sarcoma of the parotid gland—the tumour got much smaller during treatment. This was the only case of sarcoma treated long enough to test the method. It was tried in a few cases of carcinoma without any benefit resulting.

**Cystic Accessory Thyroid.**—Mr. Edmunds[6] exhibited at the Pathological Society a specimen of this form of growth. It lay some distance from the proper thyroid, which was not seen at the operation, and was apparently normal. The growth consisted of a cyst, into which projected a mass of tissue of the microscopic structure of thyroid gland. Mr. Berry and Mr. Butlin considered that these growths lying at a short distance from the thyroid were often really derived from the gland itself, though the connection could not be traced at the time of the operation.

**Round Celled Sarcoma of Tongue.**—A case is reported of a growth of this nature[7] by Dr. Dunham of New York. The tumour seemed to start from the irritation of a bite of the tongue, which caused a "blister" to form, and this became irritated by contact with decayed teeth, and after three months began to grow hard. The history of origin was like that of epithelioma, but the histological characters were distinct. The mucous membrane could be traced in the sections over the surface, and did not dip into the growth, which was composed chiefly of large round cells, but there were a few spindle shaped. The mass was traversed by delicate processes of fibrous tissue, barely sufficient in most places to furnish support to capillary vessels.

[1] Clinical Journal, Oct. 16, p. 380. [2] Lancet, Oct. 12, p. 904. [3] Wiener Klin. Rundschau, 1895, Nos. 33 and 34, and Epitome B. Med. J., Sept. 21, 1895. [4] Rif. Med., Aug. 19 and 20, 1895, and Epitome B. Med. J., Oct. 12, 1895. [5] Münich Med. Woch., Sept. 3, 1895, and Epitome B. Med. J., Oct. 12, 1895. [6] Brit. Med. J., Nov. 23, p. 1,295. [7] American Journ. Med. Sciences, Sept., 1895, p. 259.

